# LPM580098, a Novel Triple Reuptake Inhibitor of Serotonin, Noradrenaline, and Dopamine, Attenuates Neuropathic Pain

**DOI:** 10.3389/fphar.2019.00053

**Published:** 2019-02-14

**Authors:** Nannan Li, Chunmei Li, Rui Han, Yu Wang, Mina Yang, Hongbo Wang, Jingwei Tian

**Affiliations:** School of Pharmacy, Key Laboratory of Molecular Pharmacology and Drug Evaluation (Yantai University), Ministry of Education, Collaborative Innovation Center of Advanced Drug Delivery System and Biotech Drugs in Universities of Shandong, Yantai University, Yantai, China

**Keywords:** neuropathic pain, 5-HT, NE, DA, central inhibition, synaptic plasticity

## Abstract

**Background and Purpose:** Sedation and somnolence remain serious adverse effects of the existing analgesics (e.g., pregabalin, duloxetine) for neuropathic pain. The available evidence indicates that serotonin (5-HT), noradrenaline (NE), and dopamine (DA) play important roles in modulating the descending inhibitory pain pathway and sleep–wake cycle. The aim of this work was to test the hypothesis that LPM580098, a novel triple reuptake inhibitor (TRI) of 5-HT, NE, and DA, has analgesic effect, and does not induce significant adverse effects associated with central inhibition, such as sedation and somnolence.

**Methods:** The analgesic activity of LPM580098 was assessed on formalin test and spinal nerve ligation (SNL)-induced neuropathic pain model. Locomotor activity, pentobarbital sodium-induced sleeping and rota-rod tests were also conducted. *In vitro* binding and uptake assays, and Western blotting were performed to examine the potential mechanisms.

**Results:** LPM580098 suppressed the nocifensive behaviors during phase II of the formalin test in mice. In SNL rats, LPM580098 (16 mg kg^−1^) inhibited mechanical allodynia, thermal hyperalgesia and hyperexcitation of wide-dynamic range (WDR) neurons, in which the effect of LPM580098 was similar to pregabalin (30 mg kg^−1^). However, pregabalin altered the spontaneous locomotion, affected pentobarbital sodium-induced sleep, and showed a trend to perform motor dysfunction, which were not induced by LPM580098. Mechanistically, LPM580098 inhibited the uptake of 5-HT, NE, and DA, improved pain-induced changes of the synaptic functional plasticity and structural plasticity possibly via downregulating the NR2B/CaMKIIα/GluR1 and Rac1/RhoA signaling pathways.

**Conclusion:** Our results suggest that LPM580098, a novel TRI, is effective in attenuating neuropathic pain without producing unwanted sedation and somnolence associated with central nervous system (CNS) depressants.

## Introduction

Neuropathic pain represents one of the most complex chronic pain conditions that occurs during disease or nerve injury. Currently available pharmacotherapies (such as pregabalin, duloxetine, and gabapentin) that are used to alleviate neuropathic pain are often associated with significant side effects (Finnerup et al., 2015), such as sedation and somnolence. For example, somnolence reportedly is experienced after treatment with pregabalin or duloxetine, and the incidence rate is up to 15.8% and 8%, respectively (see labels). Therefore, efficacious analgesics with fewer adverse effects are urgently needed for the management of neuropathic pain.

Numerous studies suggest that antidepressants especially the SNRIs (e.g., duloxetine, venlafaxine) have demonstrated therapeutic efficacy for neuropathic pain (McCleane, 2008; Zhang et al., 2016b; Obata, 2017; Raouf et al., 2017). In particular duloxetine hydrochloride, which is a Food and Drug Administration-approved prescription drug indicated for the management of major depressive disorder, generalized anxiety disorder, diabetic peripheral neuropathic pain, fibromyalgia, and chronic musculoskeletal pain. In addition, descending dopaminergic inhibitory systems are also involved in the modulation of pain processing (Tang et al., 2009; Taniguchi et al., 2011). Interestingly, 5-HT, NE, and DA are also participated in the regulation of sleep–wake/arousal state, in which 5-HT produces bidirectional actions whereas NE and DA predominantly exert arousal role (Monti and Monti, 2007; Monti, 2011; Berridge et al., 2012). Our previous studies found that TRIs that blocked the reuptake of all three monoamine neurotransmitters showed therapeutic efficacy in animal models of depression without significant sedative effect (Tian et al., 2011; Zhang et al., 2016a). Here, we hypothesized that disrupting the reuptake of 5-HT, NE, and DA may suppress neuropathic pain without triggering unwanted side effects (i.e., sedation and somnolence).

LPM580098, 1-[2-(dimethylamino)-1-(4-phenoxyphenyl) ethyl] cyclohexanol, is a novel TRI that was designed and synthesized based on the structure of venlafaxine, a SNRI that has been shown to exert robust antidepressant activity (Skolnick and Basile, 2007) and analgesic effect (Feinberg et al., 2017). In the present study, formalin test and SNL model were used to evaluate the analgesic effect of LPM580098. In addition, we compared the side-effect profiles of LPM580098 with pregabalin using locomotor activity, pentobarbital sodium-induced sleeping, and rota-rod tests. Biochemical and histological studies were also performed to explore the potential underlying mechanisms that contribute to the analgesic effects of LPM580098.

## Materials and Methods

### Animals

Male Swiss mice (3–4 weeks old) and male Sprague Dawley rats (5–6 weeks old) were obtained from Jinan Pengyue Experimental Animal Centre (Jinan, Shandong, China). All animals were acclimated to the laboratory environment for several days before the experiment. The mice and rats were kept under a 12-h light/dark regimen with water and food given *ad libitum*. Room temperature and humidity were maintained at 21–23°C and 40–60%, respectively. All animal experiments were conducted in accordance with guidelines of the National Institutes of Health and approved by the Experimental Animal Ethics Committees of Yantai University. Animal studies were carried out in compliance with the ARRIVE guidelines (Kilkenny et al., 2010). The behavioral experimenters were kept blind from the groupings of the mice and rats in a quiet room under controlled conditions.

### Drugs and Chemicals

**FIGURE 1:**
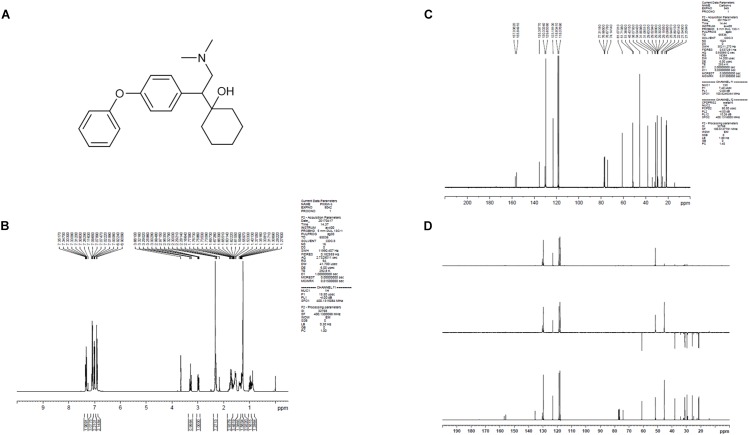
The structure information of LPM580098. **(A)** The chemical structure of LPM580098. **(B)**^1^H NMR spectra of LPM580098. **(C)** ^13^C NMR spectra of LPM580098. **(D)** Distortionless enhancement by polarization transfer spectra of LPM580098.

LPM580098 was synthesized in-house (Yantai University, Shandong, China). The procedure of synthesis was as follows: the *O*-demethylvenlafaxine, potassium methoxide solution, and methanol were mixed and placed in a water bath at 85°C in a flask. After refluxing for 60 min, the solvent was removed under reduced pressure and the residue was azeotroped with toluene three times to obtain off-white solid, which was then dissolved in *N*,*N*-dimethylformamide with iodobenzene, and cuprous iodide. The reaction mixture was heated at 152°C for 72 h under nitrogen atmosphere till the thin-layer chromatography showed that all starting material completely consumed. The solvent was removed under reduced pressure, and then extracted with dichloromethane, washed with 2% hydrochloric acid, and the combined organic phases were dried over anhydrous sodium sulfate. The residue was concentrated under reduced pressure, which was purified by silica gel column chromatography using tetrahydrofuran as the mobile phase. The obtained oil was washed with 10% NaOH aqueous solution and stirred overnight. The following brown solid precipitate was filtered, and then recrystallized from ethyl acetate. After filtration, the filter cake was collected and dried to afford off-white solid. The chemical structure of LPM580098 was shown in Figure 1A, which was confirmed by ^1^H NMR and ^13^C NMR (Figure 1B–D). The purity of the compound used in this study was checked by HPLC and found to be 99.14%. For the *in vitro* study, LPM580098 was dissolved in dimethyl sulfoxide; however, the *in vivo* study mixed LPM580098 or pregabalin capsules (Pfizer, New York, NY, United States) into 0.5% CMC-Na. Formaldehyde (Sigma, United States), pentobarbital sodium (Merck, Germany), and diazepam (Xinyi, Shandong, China) were dissolved in a saline solution.

### Radioligand Binding Assays

To determine whether LPM580098 binds to SERT, NET, or DAT, radioligand binding assays were performed as previously described (Pacholczyk et al., 1991; Pristupa et al., 1994; Tatsumi et al., 1999). CHO cell membrane homogenates (20 μg protein) were incubated with 1 nM [^3^H] nisoxetine or 4 nM [^3^H] BTCP for 120 min at 4°C, or with 2 nM [^3^H] imipramine in the absence or presence of the test compound for 60 min at 22°C. Only 10 μM of LPM580098 was performed in the experiment. Non-specific binding was determined in the presence of 1 μM desipramine, 10 μM BTCP, and 10 μM imipramine, respectively. Termination of reactions was performed by rapid filtration in vacuum using glass fiber filters (GF/B, Packard, United States) that were presoaked with 0.3% polyethylenimine, followed by several rinses in ice-cold 50 mM Tris-HCl with a 96-sample cell harvester (Unifilter, Packard, United States). Then, the filters were dried, and radioactivity was measured using a scintillation counter (Topcount, Packard, United States) and a scintillation cocktail (Microscint, Packard, United States). Specific binding was estimated as the difference between total and non-specific binding, and the results were expressed as percent inhibition of the control radioligand specific binding.

### Synaptosomal Uptake Assays

The effects of LPM580098 on the uptake of 5-HT, NE, or DA were assessed as previously described (Bolden-Watson and Richelson, 1993). Synaptosomes were prepared from the brain, hypothalamus, and striatum of rats, then incubated with 0.2 μCi [^3^H] 5-HT, 0.2 μCi [^3^H] NE or 0.2 μCi [^3^H] DA for 15 min at 37°C with or without the test or reference compound. The reactions were immediately terminated under vacuum and filtered through GF/B glass fiber filters (Packard, United States) and rinsed twice with ice-cold 50 mM Tris-HCl using a 96-sample cell harvester (Unifilter, Packard, United States). When the filters were dried, the radioactivity of each sample was measured using a scintillation cocktail (Microscint, Packard, United States) and a scintillation counter (Topcount, Packard, United States), and IC_50_ values were calculated.

### Formalin Test in Mice

The formalin model used in this study was as previously described (Tjølsen et al., 1992). Mice were allowed to habituate for at least 2 h in testing room prior to the experiment and the behaviors were conducted between 8:00 am and 1:00 pm. Mice (*n* = 10/group) were orally administered with vehicle, pregabalin (60 mg kg^−1^) or LPM580098 (16 mg kg^−1^, 32 mg kg^−1^, 64 mg kg^−1^) 60 min prior to intraplantar injection of diluted formalin solution, and acclimatized individually in custom-made Plexiglass boxes 10 min before the experiment. 20 μL of 2.5% formalin (37% formaldehyde diluted in saline) was injected into the plantar surface of the right hind paw of the mice. Observation was conducted immediately after formalin injection. The time (in seconds) spent flinching and licking the right hind paw from 0 to 5 min after injection was considered phase I, whereas phase II was the total time spent performing these responses from 15 to 40 min, and nociception was quantified based on the flinching/licking time lasted in both phases.

### Spinal Nerve Ligation (SNL) in Rats

Spinal nerve injury was induced by tight ligation of the lumbar 5 (L5) spinal nerve as described elsewhere (Kim and Chung, 1992; LaBuda and Little, 2005; Sidorova et al., 2017). Briefly, rats were anesthetized with pentobarbital sodium (35 mg kg^−1^ i.p.). Under cold-light (Olympus, Japan), the right L6 transverse process was removed, and the right L5 spinal nerve was identified then tightly ligated using a 6-0 silk suture. The SNL operation was completed by closing the muscles and skin with a 2-0 silk suture in layers. Postoperative treatments included once daily intramuscular injections of 0.4 mL penicillin (Lukang, Shandong, China) for 3 days to prevent infection or inflammation.

The experimental design was as previously described (Kim et al., 2006). In the preliminary study, we have examined the time-effect and dose-effect relationships of single administration of LPM580098 on mechanical allodynia and thermal hyperalgesia in SNL rats, the results indicate that the most appropriate point to detect analgesic effect on mechanical and thermal hypersensitivity is at 60 min after oral administration, and LPM580098 at doses of 16 mg kg^−1^ and 32 mg kg^−1^ are effective for pain treatment (see Supplementary Figure S1). We evaluate the analgesic effect of 16 mg kg^−1^ of LPM580098 at 60 min after oral administration for further studies.

**FIGURE 2:**
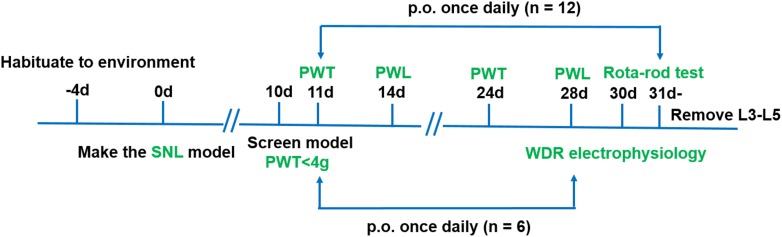
Schematic representation of the experimental design for assessing the analgesic effects of LPM580098 in a SNL model. Four groups were used in this experiment (*n* = 18/group), in which behavioral tests were performed on days 10, 11, 14, 24, 28, and 30 post-SNL surgery (*n* = 12/group), and activities of dorsal horn WDR neurons were examined on day 28 post-SNL surgery (*n* = 6/group). PWT, paw withdrawal threshold; PWL, paw withdrawal latency.

Four groups of rats with the following treatments were used in this experiment (*n* = 18/group): sham (0.5% CMC-Na), vehicle (0.5% CMC-Na), pregabalin (30 mg kg^−1^) and LPM580098 (16 mg kg^−1^). LPM580098 was daily administrated during 21 consecutive days (from days 11 to 31 post-surgery), in which behavioral tests were performed between 8:00 am and 1:00 pm on day 10, 11, 14, 24, 28, and 30 post-SNL surgery, and activities of dorsal horn WDR neurons were examined on day 28 post-SNL surgery. The timeline of the experiment is shown in Figure 2.

### Mechanical Allodynia in SNL Rats

Behavioral testing was performed on days 10, 11, and 24 post-SNL surgery. The rats were placed in a cage with a wire grid bottom (27 cm × 22 cm × 20 cm), which allowed full access to the paws, and they were allowed habituation in the test cages for 20 min. Mechanical sensory thresholds were measured by hind paw withdrawal away from a series of von Frey filaments (0.16–26 g, Bioseb, France) applied to the middle of the right hind paws. A modified Dixon ‘up-down’ technique was used in the estimation of force value (Chaplan et al., 1994).

In this study, testing was initiated using 2 g of filaments, which is in the middle of the series, delivered in a perpendicular direction into the right paw’s plantar surface for around 5 s. The stimuli were consecutively introduced, regardless of direction (ascending or descending). If no withdrawal response was observed, then the next greater force was applied; when a response was detected, a lower force was used. Mechanical allodynia in rats was assessed 60 min after oral administration by measuring the 50% PWT and calculated according to Chaplan et al. (1994).

### Thermal Hyperalgesia in SNL Rats

Thermal hyperalgesia was assessed on days 14 and 28 post-SNL surgery using a plantar testing apparatus (IITC Life Science Instruments, Model 390 G, Woodland Hills, CA, United States). The rats were placed in Perspex boxes (21 cm × 21 cm × 14 cm) on a heated glass surface and individually acclimatized for 20 min before the experiment. Thermal nociceptive thresholds were evaluated 60 min after oral administration by determining the PWL upon application of a calibrated radiant heat source. This stimulus was positioned under the center of the plantar surface of the right hind paw with the aid of an angled mirror. The infrared heat intensity was set as 46% of the maximal output of the device, and the heat source was instantly switched off at 20 s to prevent potential tissue injury. The PWL readings of five trials were averaged as the response latency.

### Electrophysiological Recording of Spinal Dorsal Horn WDR Neurons in SNL Rats

Extracellular recordings were conducted as previously described (Ding et al., 2015). Briefly, rats (*n* = 6/group) were anesthetized with urethane (1.0–1.5 g kg^−1^, i.p.) at 28 days after SNL. Tracheal cannulation was performed for ventilation, followed by the insertion of another catheter into the right carotid artery as well as jugular vein to allow continuous infusion of Tyrode’s solution. The physiological condition of the animals was monitored based on their blood pressure and rectal temperature, and was maintained within the indicated range. The lumbar enlargement involving the spinal cord was isolated by laminectomy of vertebral segments L4 and L6, and the dural spinal segments were carefully removed. A small well was created at the monitored segment to allow for the application of vehicle or drugs.

Extracellular single-unit monitoring and WDR neuron identification were performed based on their responses to brush, von Frey filaments, and pinch stimulations. Once a single unit was identified, spontaneous firing activity was determined for 5–10 min in the absence of any peripheral stimulation, and then three induced stimuli were applied to determine the receptive field and response characteristics, including (1) brush stimulus given by lightly brushing the receptive field 10 times with a camel brush, (2) calibrated von Frey filaments were given in a graded manner (1 g, 4 g, 8 g, and 15 g) 10 times with 10-s interval and 1-s on/off pattern, and (3) noxious stimulation firing induced by vascular forceps pinching receptive field that was recorded for 10 s.

### Rota-Rod Test in SNL Rats

Motor performance was assessed 30 days after SNL using an automated accelerating Rota-rod apparatus (Panlab Harvard Apparatus, Barcelona, Spain), at a velocity that gradually increased from 4 rpm to 40 rpm over a 5-min interval. Seven trials were performed (Tan et al., 2008); the first two trials were “training,” and the other five trials were consecutively performed for analysis, with a maximal time of 300 s. The latency that rats remained on the accelerating rod was recorded in seconds for all groups. Rats were given at least 2 h to acclimatize to the testing room prior to the experiment and the behaviors were conducted between 8:00 am and 1:00 pm.

### Locomotor Activity Test in Normal Rats

Rats (*n* = 10/group) were transferred into the testing room for at least 2 h prior to the test and the activity was recorded between 8:00 am and 1:00 pm with TopScan monitoring system (CleverSys Inc.) (Tian et al., 2014; Zhang et al., 2016a). Rats were orally administered with LPM580098 (16 mg kg^−1^), pregabalin (30 mg kg^−1^) or vehicle (0.5% CMC-Na) 60 min before testing; they were then put in the activity chambers (60 cm × 60 cm × 45 cm) for 10 min to habituate to the test chambers. The rats’ distance traveled (cm) in the chambers was recorded for 10 to 20 min. Chambers were cleaned using alcohol pad between animals.

### Pentobarbital Sodium-Induced Sleeping Test in Normal Mice

A pilot experiment was conducted to determine the hypnotic subthreshold dose of pentobarbital sodium (28 mg kg^−1^). Forty normal mice were then randomly divided into four groups (*n* = 10/group), in which the subthreshold dose of pentobarbital sodium was intraperitoneally injected 50 min after the administration of vehicle (0.5% CMC-Na), diazepam (3 mg kg^−1^), pregabalin (60 mg kg^−1^), and LPM580098 (32 mg kg^−1^). The sleeping states of the animals were observed for up to 30 min immediately after pentobarbital sodium injection. The number of mice that slept as evidenced by the disappearance of righting reflex for 1 min within 30 min was recorded. The test was carried out between 19:00 and 21:00.

### Dendritic Spine/Image Analysis of Spinal Dorsal Horn WDR Neurons in SNL Rats

The sham, vehicle, and LPM580098 treatment rats (*n* = 5/group) were euthanized 31 days after SNL without fixation for Golgi-cox staining using a FD Rapid GolgiStain^TM^ kit (FD Neuro Technologies, Inc.). Briefly, fresh tissues of the lumbar enlargement (L3–L5) of the spinal cord were immediately collected, washed with deionized water, and then immersed in infiltration solutions. Following the manufacturer’s guidelines, 180-μm thick tissue sections were prepared using a freezing microtome, which were then mounted on gelatin-coated glass slides. The sections on the slides were stained, rinsed in deionized water, dehydrated through an ethanol gradient, cleared in xylene, and coverslipped with Permount^TM^ mounting medium.

Tissue sections of the Golgi-stained coronal spinal cord were evaluated by light microscopy (Nikon, Japan). For spine analysis, specific criteria in screening dendritic segments were employed (Crupi et al., 2016), which facilitated the identification of cells that showed similar morphological features as those in WDR neurons (Tan et al., 2008). Dendritic segments were imaged with a 100× oil immersion objective. A total of 75 neurons (*n* = 25 neurons from five rats in each group) in spinal dorsal horn were included in our analysis.

Dendritic spines were identified based on specific morphological characteristics, and protrusions from dendritic branches with distinct necks were considered as spines (Golden et al., 2013). The dendritic spines were further classified as thin and mushroom-shaped, and spine density was presented as spines per 10-μm dendritic length in our research.

### Tissue Preparation and Western Blot in SNL Rats

The spinal dorsal horn of the L3–L5 lumbar enlargement segments were freshly removed under anesthesia 31 days after SNL, snap frozen in dry ice, and stored at −150°C. For total protein analysis, the tissues were homogenized in RIPA buffer combined with a proteinase inhibitor phenylmethylsulfonyl fluoride (PMSF) (100:1) (Li et al., 2018; Yang et al., 2018). Portions of tissue lysate were centrifuged (12,000 rpm, 20 min, 4°C), and then supernatant was collected for subsequent protein quantification using a BCA protein assay kit (Beyotime, Shanghai, China). For membrane protein analysis, the tissues were homogenized in a Membrane Protein Extraction Kit A combined with a proteinase inhibitor PMSF (100:1). The samples were centrifuged (700 × *g*, 10 min, 4°C) to separate nuclear enriched pellet (P1) and the supernatant (S1) fraction. The S1 fraction was then centrifuged (14,000 × *g*, 30 min, 4°C) to isolate the pellet (P2; crude membranes). We resuspended the P2 in Membrane Protein Extraction Kit B, followed by vortexing (10 s) and then laid on ice (5–10 min); these steps were repeated twice. Then, the samples were centrifuged (14,000 × *g*, 5 min, 4°C) to obtain the S3, and a BCA protein assay kit was used to determine protein concentrations.

Equal amounts of total protein samples (50 μg lane^−1^) were loaded onto SDS- polyacrylamide gel (4–20%, GenScript, Nanjing, China). The separated proteins were immunoblotted on to polyvinylidene fluoride membranes (Millipore, MA, United States), incubated in 5% skimmed dry milk for 2 h at room temperature, and then incubated overnight with primary antibodies at 4°C. Then, the immunoblots were rinsed and allowed to hybridize with horseradish peroxidase-conjugated secondary antibodies of the same species source for 2 h at room temperature. The expression of proteins was detected using an enhanced chemiluminescence kit (Beyotime, Shanghai, China). Western blots were analyzed using ImageJ software.

The following primary antibodies were used: rabbit anti-Rac1 plus Cdc42 phosphoSer71 (1:1000, Abcam, Cambridge, United Kingdom, Cat# ab5482), mouse anti-Rac1 (1:1000, Abcam, Cambridge, United Kingdom, Cat# ab33186), mouse anti-PSD-95 (1:1000, Abcam, Cambridge, United Kingdom, Cat# ab192757), rabbit anti-synaptophysin (1:2000, Abcam, Cambridge, United Kingdom, Cat# ab32127), rabbit anti-RhoA (1:1000, Abcam, Cambridge, United Kingdom, Cat# ab187027), rabbit anti-RhoA phosphoSer188 (1:1000, Abcam, Cambridge, United Kingdom, Cat# ab41435), mouse anti-CaMKII alpha (1:1000, Cell Signaling Technology, MA, United States, Cat# 50049), rabbit anti-CaMKII alpha phosphoThr286 (1:1000, Abcam, Cambridge, United Kingdom, Cat# ab5683), rabbit anti-glutamate receptor 1 (1:1000, Abcam, Cambridge, United Kingdom, Cat# ab31232), rabbit anti-glutamate receptor 1 phosphoSer831 (1:1000, Abcam, Cambridge, United Kingdom, Cat# ab109464), rabbit anti-NMDAR2B (1:1000, Abcam, Cambridge, United Kingdom, Cat# ab65783), rabbit anti-NMDAR2B phosphoSer1303 (1:1000, Abcam, Cambridge, United Kingdom, Cat# ab81271), rabbit anti-NMDAR2B phosphoTyr1472 (1:1000, Abcam, Cambridge, United Kingdom, Cat# ab3856), mouse anti-β-actin (1:1000, Beyotime, Shanghai, China), mouse anti-GAPDH (1:1000, Beyotime, Shanghai, China). The following secondary antibodies were used: goat anti-mouse HRP (1:2000, Beyotime, Shanghai, China) and goat anti-rabbit HRP (1:2000, Beyotime, Shanghai, China).

### Data and Statistical Analysis

The data and statistical analysis comply with the recommendations on experimental design and analysis in pharmacology (Curtis et al., 2018). All data showing *p* < 0.05 were considered statistically significant for all analyses, using either parametric or non-parametric approaches, including one-way analysis of variance (ANOVA) followed by Dunnett’s *post hoc* test, un-paired Student’s *t*-test, and Mann–Whitney test. Data was analyzed using IBM SPSS Statistics version 19.0 (Chicago, IL, United States), GraphPad Prism version 5.0 (San Diego, CA, United States) and presented as the mean ± SEM.

## Results

### *In vitro* Binding Profiles of LPM580098

To determine whether LPM580098 was capable of binding the SERT, NET, and DAT, we performed the *in vitro* binding assays. In our study, LPM580098 (10 μM) displayed significant binding affinities at the transporters of 5-HT, NE, and DA, in which the inhibitory efficiency reached 90.3%, 45.0%, and 77.5%, respectively.

### Inhibitory Effect of LPM580098 on 5-HT, NE, and DA Uptake

**FIGURE 3:**
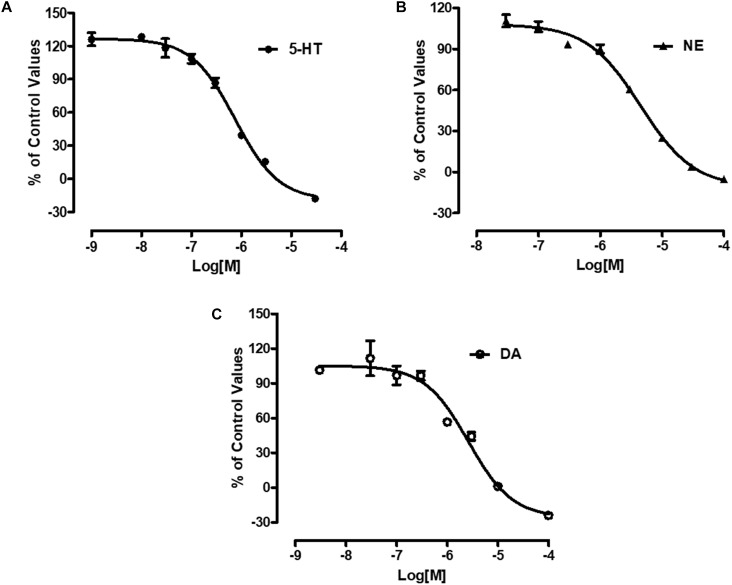
Effects of LPM580098 on the uptake of 5-HT, NE, and DA. **(A)** 5-HT, **(B)** NE, and **(C)** DA were expressed as percent inhibition of control specific activity (*n* = 3, the number of independent experiments).

Radiolabeled neurotransmitters uptake assays were used to determine the effects of LPM580098 on 5-HT, NE, or DA uptake. LPM580098 effectively blocked ^[3H]^ 5-HT, ^[3H]^ NE or ^[3H]^ DA uptake into the rat synaptosomes in a dose-dependent manner. The IC_50_ values for inhibiting 5-HT, NE, and DA uptake were 0.89 ± 0.13 μM, 3.94 ± 0.31 μM, and 1.63 ± 0.39 μM, respectively (Figure 3; see Supplementary Table S1).

### LPM580098 Reduced Formalin-Induced Nociceptive Behavior in Mice

**FIGURE 4:**
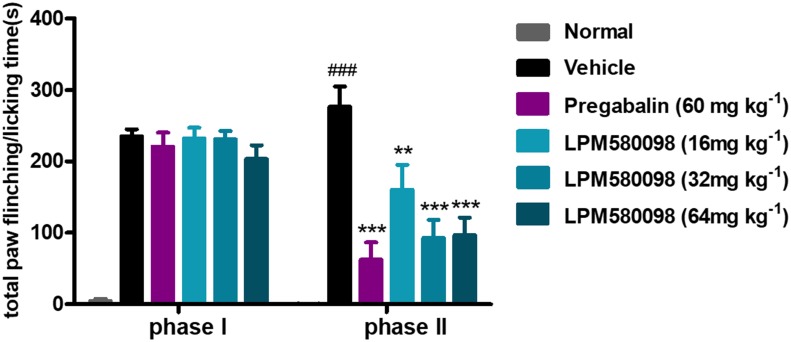
Effects of LPM580098 on formalin-induced pain behaviors in mice. Pregabalin (60 mg kg^−1^) and LPM580098 (16 mg kg^−1^, 32 mg kg^−1^, and 64 mg kg^−1^) significantly suppressed formalin-induced nociceptive behaviors during phase II (15–40 min) but not during phase I (0–5 min). Data were expressed as the mean ± SEM, *n* = 10/group. ^###^*p* < 0.001 versus normal group, ^∗∗^*p* < 0.01 and ^∗∗∗^*p* < 0.001 versus vehicle group, one-way ANOVA followed by Dunnett’s *post hoc* test.

Spontaneous nociceptive behaviors due to noxious chemical stimuli were tested using the formalin test, which induced time-dependent biphasic responses. To assess the potential central analgesic activity of LPM580098, the total time spent on flinching and licking the injected paw was analyzed, in which during phase I (0–5 min), the time apparently did not reduce in pregabalin (60 mg kg^−1^) and LPM580098 (16 mg kg^−1^, 32 mg kg^−1^, and 64 mg kg^−1^) groups, whereas in phase II (15–40 min), LPM580098 produced a marked and dose-related inhibition in formalin-induced nociceptive behaviors (*p* < 0.01, *p* < 0.001, *p* < 0.001, respectively). Pregabalin also significantly attenuated pain behaviors compared to the vehicle-treated mice (Figure 4, *p* < 0.001; see Supplementary Table S1).

### LPM580098 Attenuated Pain-Related Behaviors in SNL Rats

To further evaluate the analgesic effect of LPM580098, an SNL-induced neuropathic pain model in rats was established and all rats were tested for mechanical allodynia and thermal hyperalgesia.

**FIGURE 5:**
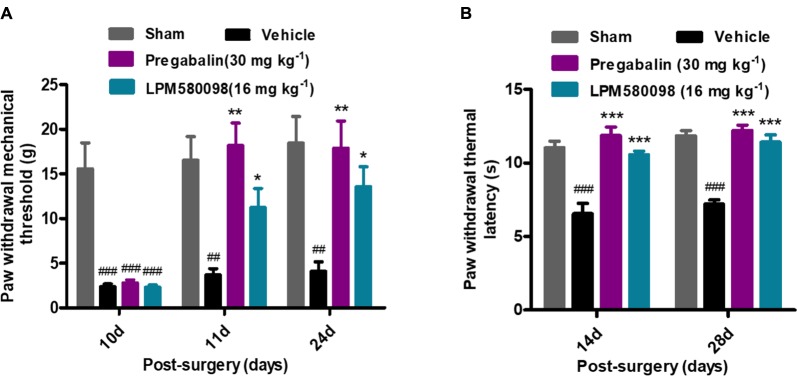
Effects of LPM580098 on SNL-induced pain behaviors: **(A)** mechanical allodynia, **(B)** thermal hyperalgesia. After SNL, the stimulus threshold for right-hind paw withdrawal was reduced in response to mechanical and thermal stimulus intensities. LPM580098 (16 mg kg^−1^) and pregabalin (30 mg kg^−1^) increased mechanical threshold and thermal latencies at 60 min after administration compared to the SNL-vehicle group, however, the analgesic effect of pregabalin was somewhat more effective than LPM580098. Graphs were expressed as the mean ± SEM, *n* = 12/group. ^##^*p* < 0.01, ^###^*p* < 0.001 versus sham group, ^∗^*p* < 0.05, ^∗∗^*p* < 0.01, ^∗∗∗^*p* < 0.001 versus vehicle group, one-way ANOVA followed by Dunnett’s *post hoc* analysis.

After SNL surgery, mechanical PWT in the right hind paw significantly decreased in the SNL-vehicle group compared to the sham animals (*p* < 0.001). As shown in Figure 5A; see Supplementary Table S1, both LPM580098 (16 mg kg^−1^) and pregabalin (30 mg kg^−1^) treatment showed significantly increased pain threshold on days 11 and 24 after SNL compared to rats that received the vehicle (*p* < 0.05, *p* < 0.01, respectively). No statistical difference was observed between pregabalin and LPM580098 groups despite the trend that pregabalin was somewhat more effective than LPM580098 at this dose (*p* > 0.05).

Thermal PWL of the SNL-vehicle rats were markedly decreased from 11.04 ± 0.45 s to 6.56 ± 0.70 s, demonstrating the development of significant thermal hyperalgesia in the right hind paw. LPM580098 (16 mg kg^−1^) and pregabalin (30 mg kg^−1^) significantly attenuated the thermal hyperalgesia on days 14 and 28 (*p* < 0.001, *p* < 0.001, respectively) post-SNL surgery with similar effectiveness (*p* > 0.05, Figure 5B; see Supplementary Table S1). These results indicated that LPM580098 attenuated mechanical allodynia and thermal hyperalgesia in SNL rats, and the analgesic effect is similar to pregabalin.

### LPM580098 Reduced SNL-Induced Hyperexcitation in Spinal Dorsal Horn WDR Neurons

**FIGURE 6:**
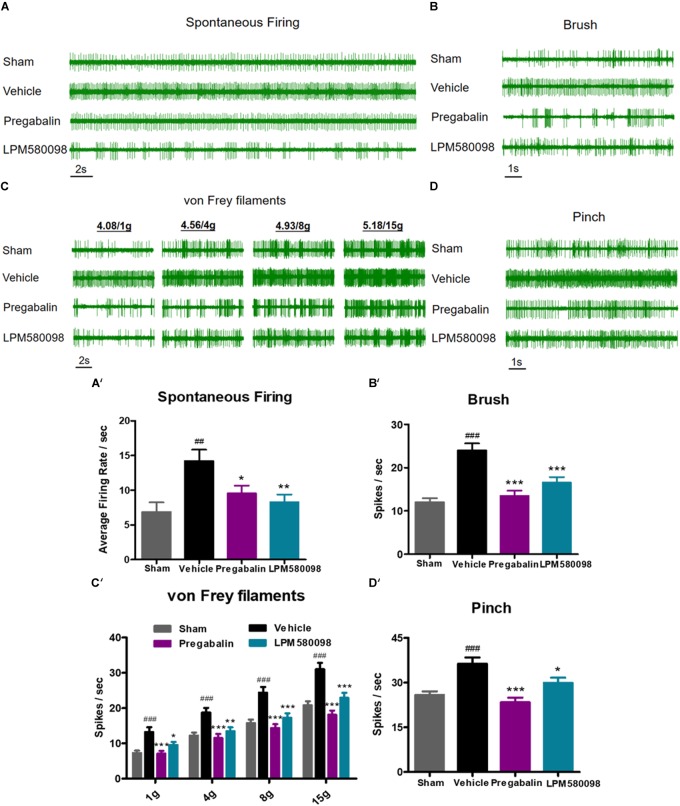
SNL-induced hyperexcitation is reduced by LPM580098 treatment in WDR neurons. **(A–D)** Representative extracellular recording raw traces of WDR neurons activity in response to spontaneous firing, brush, von Frey filaments and pinch 1 h after administration. **(A′–D′)** The spike frequency of WDR neurons to spontaneous firing and mechanical stimulation (brush, von Frey filaments, and pinch) in the sham, vehicle, pregabalin, and LPM580098 groups, respectively. Data were presented as the mean ± SEM, *n* = 6 rats per group. ^##^*p* < 0.01, ^###^*p* < 0.001 versus sham group, ^∗^*p* < 0.05, ^∗∗^*p* < 0.01, ^∗∗∗^*p* < 0.001 versus vehicle group, one-way ANOVA followed by Dunnett’s *post hoc* test.

To further determine the effects of LPM580098 and pregabalin on SNL-induced hyperexcitation in WDR neurons, *in vivo* extracellular recording was conducted at 28 days post-SNL surgery. Representative extracellular recordings of the WDR neurons in response to spontaneous firing and mechanical stimulation (brush, von Frey filaments, and pinch) are shown in Figure 6A–D. We observed a significant increase in the SNL-induced neuronal spontaneous firing rate (*p* < 0.01, *n* = 21 neurons), which was reduced by LPM580098 (16 mg kg^−1^) or pregabalin (30 mg kg^−1^) treatment (*p* < 0.01, *n* = 25 neurons; *p* < 0.05, *n* = 20 neurons) (Figure 6A′; see Supplementary Table S1). Furthermore, the number of spike responses of neurons of the spinal WDR in response to mechanical stimulation (brush, von Frey filaments, and pinch) was significantly decreased by LPM580098 (brush, *p* < 0.001, *n* = 43 neurons; von Frey filaments, 1 g, *p* < 0.05, 4 g, *p* < 0.01, 8 g and 15 g, *p* < 0.001, *n* = 43 neurons; pinch, *p* < 0.05, *n* = 43 neurons) or pregabalin (brush, *p* < 0.001, *n* = 39 neurons; von Frey filaments, 1 g, 4 g, 8 g, and 15 g, *p* < 0.001, *n* = 39 neurons; pinch, *p* < 0.001, *n* = 39 neurons), and the effects were similar in both groups (brush, *p* > 0.05; von Frey filaments, *p* > 0.05; pinch, *p* > 0.05, Figure 6B′–D′; see Supplementary Table S1).

### LPM580098 Did Not Impair Motor Performance in SNL Rats

**FIGURE 7:**
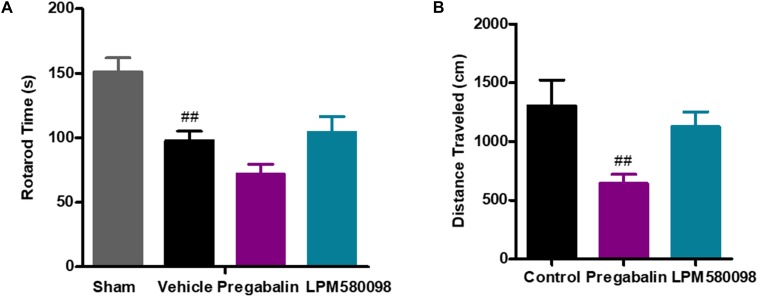
Effects of LPM580098 on **(A)** rota-rod test in SNL rats, and **(B)** locomotor activity in normal rats. Rats were single administered with vehicle (0.5% CMCN-Na), pregabalin (30 mg kg^−1^) or LPM580098 (16 mg kg^−1^) 60 min before test. One hour later, motor performance or locomotor activity were recorded. Pregabalin decreased the spontaneous locomotion of rats, and showed a trend to reduce rota-rod fall latency, which were not seen with LPM580098. Graphs were presented as mean ± SEM, *n* = 12, 10 animals, respectively. ^##^*p* < 0.01 versus sham group,^##^*p* < 0.01 versus control group, one-way ANOVA followed by Dunnett’s *post hoc* test.

To test whether the dose of LPM580098 used in the pain studies impaired the motoric behavior in rats, rota-rod test was performed on day 30 after SNL. The sham rats could remain on the rota-rod for up to 151.20 ± 10.76 s, whereas the SNL rats demonstrated impaired performance (97.85 ± 7.24 s, *p* < 0.01). Pregabalin (30 mg kg^−1^) showed a trend to reduce the retention time on rota-rod compared to vehicle treatment (71.76 ± 7.70 s, *p* > 0.05), while LPM580098 (16 mg kg^−1^) did not cause difference relative to vehicle treatment (104.17 ± 12.37 s, *p* > 0.05, Figure 7A; see Supplementary Table S1).

### LPM580098 Did Not Alter Locomotor Activity in Normal Rats

To test whether the dose of LPM580098 used in the pain studies produced the sedative effect, we performed the locomotor activity test in normal rats. Single administration of LPM580098 (16 mg kg^−1^) did not produce a statistically significant increase/decrease in locomotor activity (*p* > 0.05), while pregabalin (30 mg kg^−1^) treatment in this study significantly reduced the spontaneous activity in rats compared to control animals (*p* < 0.01, Figure 7B; see Supplementary Table S1).

### LPM580098 Did Not Enhance the Sleep-Inducing Effect of the Subthreshold Dose of Pentobarbital Sodium in Normal Mice

**FIGURE 8:**
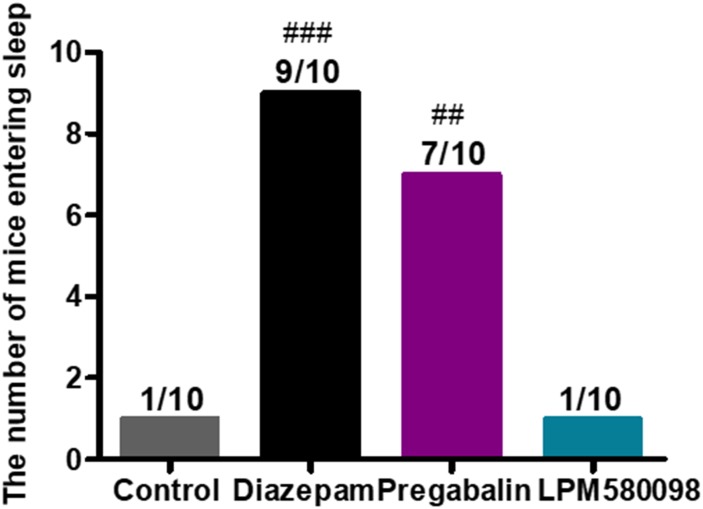
Effect of LPM580098 on sleeping onset treated by subthreshold dose of pentobarbital sodium in normal mice. Pentobarbital sodium (28 mg kg^−1^) was intraperitoneally injected 50 min after administration of vehicle (0.5% CMC-Na), diazepam (3 mg kg^−1^), pregabalin (60 mg kg^−1^), and LPM580098 (32 mg kg^−1^). The sleep states of animals were observed within 30 min after injection and the number of sleeping mice within 30 min was recorded, *n* = 10/group, ^##^*p* < 0.01, ^###^*p* < 0.001 versus control group, non-parametric approach was used followed by Mann–Whitney test.

In the present study, pentobarbital sodium-induced sleeping test was used in normal mice to evaluate whether LPM580098 had sleep-promoting effects. In this study, we observed that LPM580098, unlike pregabalin, did not play a hypnotic role. As shown in Figure 8; see Supplementary Table S1, only one mouse in vehicle group and LPM580098-single treated (32 mg kg^−1^) animals demonstrated loss of righting reflex, whereas seven animals in the pregabalin group (60 mg kg^−1^) and nine mice in the diazepam group (3 mg kg^−1^) showed loss of righting reflex (*p* < 0.01, *p* < 0.001, respectively).

### Effects of LPM580098 on Dendritic Spines Remodeling in the Dorsal Horn After SNL

**FIGURE 9:**
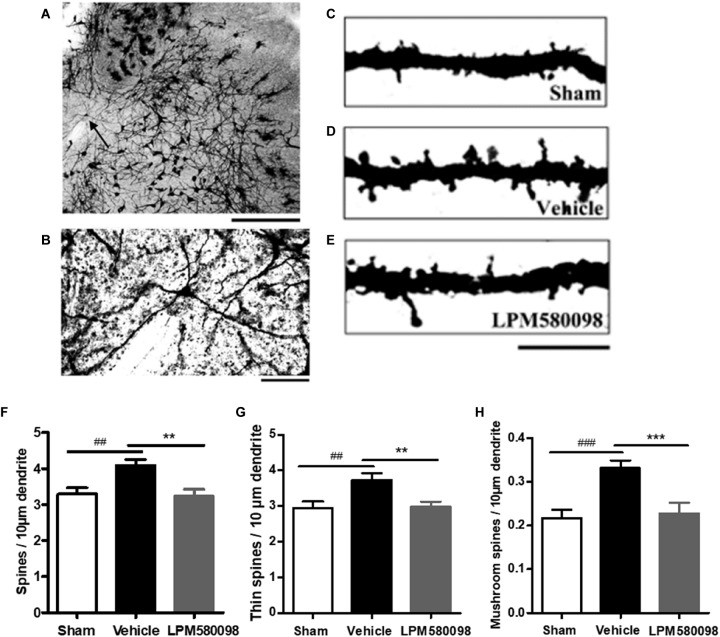
Dendritic spines were examined in Golgi-stained coronal tissue sections of the spinal cord in SNL rats. **(A)** A representative WDR neuron of the dorsal horn. **(B)** Magnified image of the neuron shown in **(A)**. **(C)** A dendritic segment from the sham group. **(D)** Approximately 31 days after SNL, a dendritic branch segment showed an increase in spine density. **(E)** Treatment with LPM580098 significantly reduced the density of spines after SNL. All dendritic spines from each group were assessed in terms of **(F)** total spine density **(G)** thin spine density, and **(H)** mushroom spine density. The SNL-vehicle group showed an increase in total spine density, thin spine density, and mushroom spines density compared to the sham animals, whereas these increase were reduced with LPM580098 treatment. Scale bars: **(A)** 500 μm, **(B)** 100 μm, **(C–E)** 10 μm. Values were mean ± SEM. (sham: *n* = 25 neurons; vehicle, *n* = 25 neurons; LPM580098, *n* = 25 neurons). ^##^*p* < 0.01, ^###^*p* < 0.001 versus sham group, ^∗∗^*p* < 0.01, ^∗∗∗^*p* < 0.001 versus vehicle group, one-way ANOVA followed by Dunnett’s *post hoc* test.

To determine whether LPM580098 treatment inhibits dendritic spine remodeling of WDR neurons after SNL, L3-L5 segments of the spinal cord were freshly removed 31 days after SNL and then the Golgi-stained neurons of each group were evaluated. A representative neuron and three dendritic segments were shown in Figure 9A–E. Dendritic spine density was assessed as spines per 10-μm dendritic length. Figure 9F; see Supplementary Table S1 showed that the total spine density was significantly increased after SNL surgery compared to the sham animals (*p* < 0.01), SNL-induced increase in total spine density was decreased by LPM580098 (*p* < 0.01). The density of thin (Figure 9G; see Supplementary Table S1) and mushroom-shaped spines (Figure 9H; see Supplementary Table S1) were also examined. After SNL surgery, the thin-shaped spine density was significantly increased compared to the sham animals (*p* < 0.01) and LPM580098 treatment induced a marked decrease in thin spine density relative to the SNL group (*p* < 0.01). The density of mushroom spines increased 52.72% after SNL surgery compared to the sham animals (*p* < 0.001); LPM580098 treatment markedly reduced the mushroom-shaped spine density (*p* < 0.001) relative to that observed in the SNL group. These results showed that LPM580098 improved dendritic spine remodeling of WDR neurons after SNL.

### Effects of LPM580098 on Synaptic Plasticity-Associated Proteins in the Spinal Dorsal Horn After SNL

**FIGURE 10:**
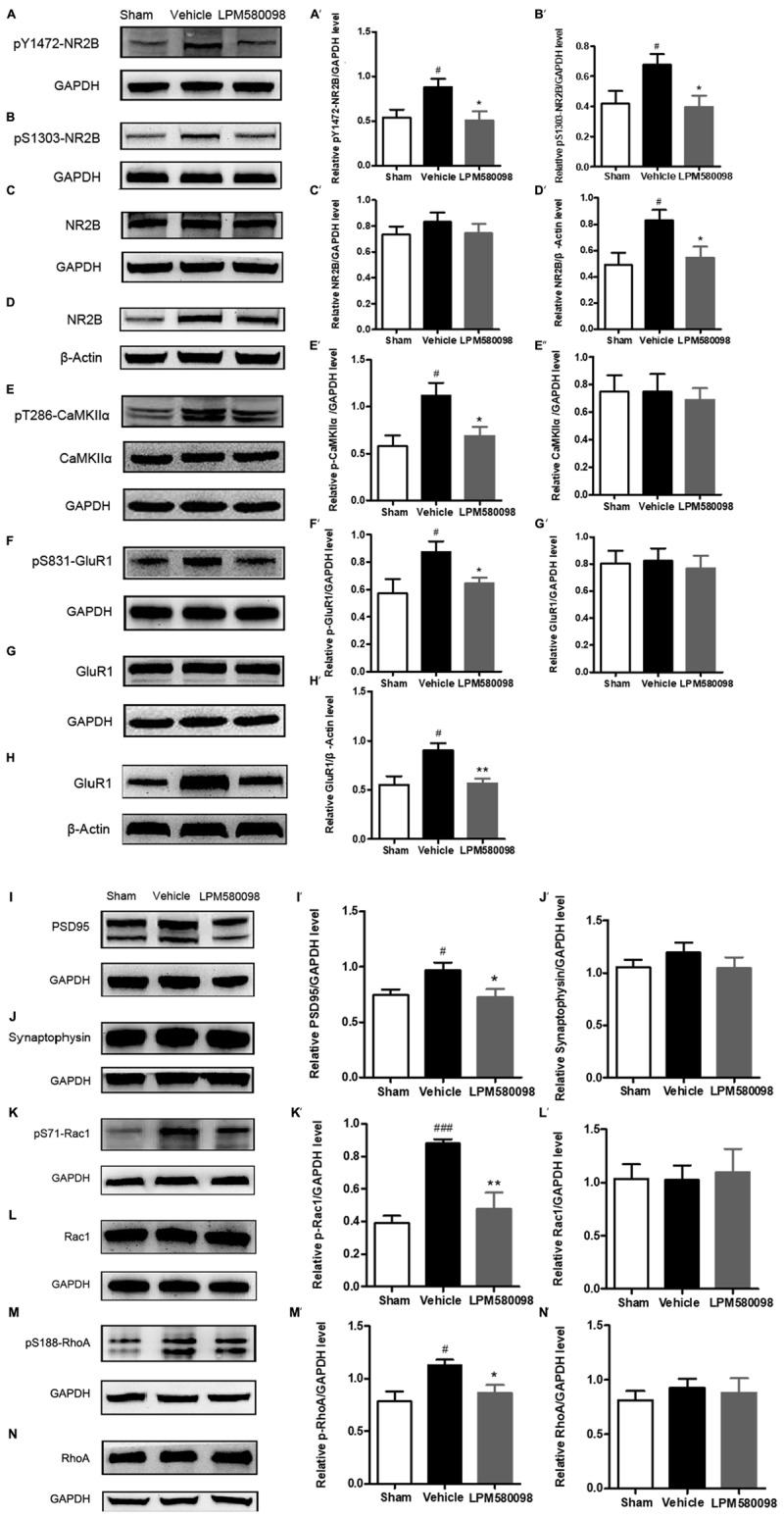
Effects of LPM580098 on synaptic plasticity-associated proteins in the spinal dorsal horn after SNL. **(A–C, E–G, I–N)** were extracted from total protein S1; **(D,H)** were extracted from membrane fraction S3. The expression of **(A)** pY1472-NR2B, **(B)** pS1303-NR2B, **(D)** NR2B, **(E)** pT286-CaMKIIα, **(F)** pS831-GluR1, **(H)** GluR1, **(I)** PSD95, **(K)** pS71-Rac1, and **(M)** pS188-RhoA proteins significantly increased after SNL compared to the sham controls, whereas those was significantly decreased after LPM580098 treatment relative to the SNL group. No significant differences in total **(C)** NR2B, **(E)** CaMKIIα, **(G)** GluR1, **(J)** synaptophysin, **(L)** Rac1, and **(N)** RhoA levels were observed among groups. Quantification was performed on five blots for each protein (**A′**–**N′**). Graphs were plotted as mean ± SEM. ^#^*p* < 0.05, ^###^*p* < 0.001 versus sham group, ^∗^*p* < 0.05, ^∗∗^*p* < 0.01 versus vehicle group. Un-paired Student’s *t*-test was used to assess statistical difference in the band intensity.

After SNL surgery, the frequency of neuronal depolarization was enhanced and the concentration of intracellular Ca^2+^ increased. To determine the underlying molecular mechanism of the effect of LPM580098 on the enhanced synaptic transmission and subsequent pain modulation in the spinal cord after nerve injury, fresh spinal dorsal horns were prepared 31 days after SNL for Western blot analysis. To figure out whether LPM580098 downregulated the synaptic plasticity-associated proteins after SNL, we examined the expression levels of NMDAR 2B subunit (NR2B), glutamate receptor (GluR1), CaMKIIα, and their corresponding phosphorylation sites, and found a significant increase in the expression levels of phosphorylated NR2B subunit (Tyr1472, Ser1303) (Figure 10A,B), GluR1 subunit (Ser831) (Figure 10F), and CaMKIIα (Thr286) (Figure 10E) after SNL compared to the sham controls, whereas this increase was significantly decreased after LPM580098 treatment. No significant differences in the total expression levels of NR2B, GluR1, and CaMKIIα were observed among all groups (Figure 10C,E,G).

To determine whether LPM580098 treatment attenuates the membrane translocation in NR2B and GluR1 after neuropathic pain, we further examined the expression levels of GluR1 and NR2B subunits in the membrane fraction and found that both of them were significantly increased in the SNL group whereas this increase was significantly decreased by LPM580098 (Figure 10D,H).

We also examined synapse-associated protein (postsynaptic density protein PSD95 and synaptophysin). The expression of PSD95, a postsynaptic marker related to NMDAR and AMPAR, was significantly increased in SNL tissues and LPM580098 attenuated this increase (Figure 10I). Synaptic-vesicle associated synaptophysin was used as a presynaptic indicator of synaptogenesis. Only a slight and insignificant increase in synaptophysin level was observed after SNL surgery (Figure 10J).

To investigate the underlying molecular mechanism of LPM580098 on synaptic structural plasticity, we analyzed the expression levels of endogenous phosphorylated Rac1 and RhoA, two members of small GTPase family. Western blot analyses indicated a significantly higher level of activated Rac1 and RhoA in the SNL group compared to the control levels and LPM580098 treatment reduced the increased protein levels (Figure 10K,M). No differences in the levels of total Rac1 and RhoA were noted among the different groups (Figure 10L,N).

## Discussion

**FIGURE 11:**
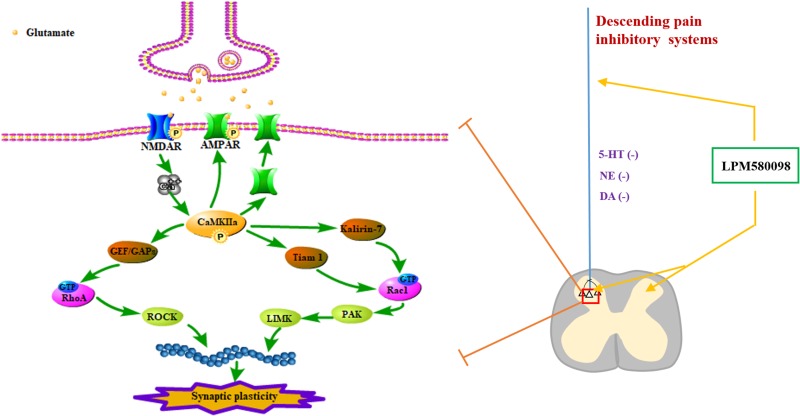
Signaling pathways involved in regulating neuropathic pain using LPM580098. LPM580098 increases 5-HT, NE, and DA by blocking SERT, NET, and DAT at the terminal of the descending serotonergic, noradrenergic, and dopaminergic systems, and these transmitters may affect the release of presynaptic glutamate in the spinal dorsal horn, which can activate postsynaptic NMDAR, thereby leading to the influx of Ca^2+^. NMDAR-dependent Ca^2+^ influx activates CaMKIIα, which in turn regulates the activity of AMPAR and increases its insertion in membranes. Other signaling cascades involving the Rho family of small GTPases (Rac1, RhoA) modulate spine morphology by remodeling the actin cytoskeleton.

Neuropathic pain is a debilitating condition and often difficult to treat, which significantly influences patients’ social function and quality of life. Clinically used first-line drugs for the treatment of neuropathic pain (e.g., pregabalin, duloxetine) only have limited therapeutic efficacy but come with serious side effects such as sedation and somnolence. It is well established that central monoaminergic system (i.e., 5-HT, NE, and DA) plays a crucial role in sleep–wake regulation and modulation of pain processing, here we investigated the effects and its potential mechanism of a novel TRI LPM580098 for the first time. Our results demonstrated that LPM580098 (16 mg kg^−1^) produced marked analgesic activity to a level that was similar to pregabalin (30 mg kg^−1^) without producing common side effects such as sedation and somnolence. Mechanistically, LPM580098 disrupted the presynaptic reuptake of 5-HT, NE, and DA, which then presumably inhibited synaptic transmission via the NR2B/CaMKIIα/GluR1 pathway, as well as prevented the synaptic structure remodeling by the regulation of Rac1/RhoA expression (Figure 11).

Formalin test is a commonly used persistent pain model that typically demonstrates two temporally different phases. Phase I begins immediately after the injection of formalin and reflects primary afferent activation due to direct chemical stimulation of peripheral nociceptors, whereas phase II is dependent on peripheral inflammatory responses as well as functional modifications of central sensitization (Tjølsen et al., 1992). Here, we first examined the potential analgesic effect of LPM580098 using formalin test in mice given the fact that this was a rapid and extensively validated assay for determining analgesic activity. We found that acute LPM580098 treatment was efficacious in reducing the total time spent on flinching and licking the injected paw during phase II. This initial testing suggests that LPM580098 may be able to enter the CNS and induce centrally mediated analgesic effects.

To further examine the potential analgesic effect of LPM580098, an SNL-induced neuropathic pain model in rats was established, and behavioral tests clearly demonstrated that oral administration of LPM580098 (16 mg kg^−1^) induced significant attenuation in both mechanical allodynia and thermal hyperalgesia. This level of analgesia was similar to that induced by pregabalin (30 mg kg^−1^). The electrophysiological activity of spinal WDR neurons is a suitable *in vivo* model for pain assessment, including hyperexcitability (Giordano, 2005; Baron et al., 2010). Here, we found that LPM580098 (16 mg kg^−1^) significantly attenuated the hyperexcitability of WDR cells, and the effectiveness was similar to that of pregabalin (30 mg kg^−1^). Together, these results clearly show that LPM580098 (16 mg kg^−1^) produces significant analgesic effect and the effectiveness is similar to pregabalin (30 mg kg^−1^) on neuropathic pain in rats.

To evaluate the potential sedative and somnolence effects of LPM580098, several behavioral tests were performed, including the locomotor activity, rota-rod test, and pentobarbital sodium-induced sleeping test. We found that pregabalin at a dose that was effective in producing analgesic activity (30 mg kg^−1^) decreased the spontaneous locomotion, and showed a trend to reduce rota-rod fall latency in rats, which were not seen with LPM580098 (16 mg kg^−1^). In the pentobarbital sodium-induced sleeping test in mice, pregabalin (60 mg kg^−1^) enhanced the sleep-inducing effect of the subthreshold dose of pentobarbital sodium whereas LPM580098 (32 mg kg^−1^) had no effect. Combined, these findings demonstrate that the apparent analgesic effect of pregabalin in rats could be partly attributed to its sedation and somnolence effects, while LPM580098 displays specific anti-nociceptive effect without producing sedation and somnolence.

Because, we observed marked analgesic effects of LPM580098 in several animal pain measures, we subsequently examined the potential molecular mechanisms that may help explain the analgesic effects of LPM580098.

It is reported that neuropathic pain has been linked to dysfunction of the pain descending inhibitory pathways of the CNS. Descending modulatory system arising with incoming nociceptive signal from the cortex, hypothalamus amygdala and pretectal nucleus finally feeds down to the spinal dorsal horn to affect further inputs into the spinal cord, causing the feedback circuit to continue, in which 5-HT, NE, and DA are key monoamines involved in the modulation of pain descending inhibitory pathways (Bannister et al., 2009; Ossipov et al., 2010; Lopez-Alvarez et al., 2018). Descending 5-HT pathway from the rostral ventromedial medulla, which has 5-HT-rich nucleus raphe magnus at its core, passes down neuronal signals to the dorsal horn, exerts pro- and anti-nociceptive actions on pain processing, in which the anti-nociceptive effects of brainstem 5-HT are largely mediated by spinal 5-HT_1A_ receptors (Bannister et al., 2009; Viguier et al., 2013). NE is sourced from brainstem nuclei (A1–A7), especially in locus ceruleus, A7 and A5 regions, that convey nociceptive signals to spinal loci in a similar manner as the descending 5-HT system. Descending NE pathway directly inhibits nociceptive transmission mainly through activation of α2-adrenergic receptors in the dorsal horn (Millan, 2002; Lu et al., 2010). Originating from the A11 region, descending DA pathway also plays an important role in the process of anti-nociception in multiple regions of the CNS, including the basal ganglia, thalamus, insular cortex, anterior cingulate cortex, periaqueductal gray, and spinal cord (Taniguchi et al., 2011; Jarcho et al., 2012; Li et al., 2016), D_2_-like receptor activation may prevent the inhibitory action of GABAergic interneurons on output neurons, leading to activation of the brainstem descending inhibitory system and depression of nociceptive inputs in the spinal dorsal horn level.

Of note, 5-HT, NE, and DA are also involved in the regulation of sleep–wake cycle. The dorsal raphe nucleus provides the principal source of 5-HT innervation in the control of sleep–wake state. The effect of 5-HT on sleep–wake behavior depends upon the degree to which the serotonergic system is activated, in which facilitatory effect in the regulation of wake is mainly via 5-HT_1A_, 5-HT_1B_ receptors (Monti, 2011). The locus coeruleus that provides the majority of brain NE exerts potent wake-promoting actions by acting on activation of noradrenergic α and β receptors (Berridge et al., 2012). In addition, DA-containing neurons involved in the regulation of sleep and waking arise in the VTA and the SNc, in which activation of postsynaptic D_1−_ receptors has a facilitatory role in the modulation of behavioral arousal and stimulation of D_2−_ receptors expressed by GABAergic neurons that synapse with cells located in structures involved in the occurrence of waking, including the basal forebrain and the cerebral cortex, also promote the occurrence of arousal systems (Monti and Monti, 2007). The available evidence suggested that during wake, there occurs an increase of burst firing activity of DA neurons, and an enhanced release of DA in the VTA, SNc, and a number of forebrain structures.

In the present study, we found that LPM580098 bound to SERT, NET, and DAT, and inhibited the presynaptic reuptake of 5-HT, NE, and DA. Results suggest that the potential analgesic mechanisms of LPM580098 are due to the presynaptic reuptake inhibition of 5-HT, NE and DA in pain descending modulatory system, thereby increasing their concentrations in synaptic cleft and then inhibit post-synaptic neurotransmission (including glutamate) in the spinal dorsal horn. However, LPM580098 does not induce sedation and somnolence, which may be largely attributed to the presynaptic reuptake inhibition of DA, and partly 5-HT and NE reuptake inhibition in brain regions associated with sleep–wake cycle, considering that the NE and 5-HT reuptake inhibitor duloxetine produces significant sedation and somnolence.

An important property of chronic pain regulation is synaptic plasticity. Synaptic plasticity, including functional plasticity and structural plasticity, may explain how the nervous system modifies its neuronal circuits to adapt, modify, and keep information. These dynamic synaptic alterations are involved in the spinal dorsal horn during pain processing, in which synaptic functional plasticity reflects long-lasting potentiation of synaptic transmission. In chronic pain states, the efficiency of glutamate-mediated excitatory synaptic transmission is increased, which leads to more neuronal depolarization, activation of NMDAR and AMPAR, and an increase in intracellular Ca^2+^ levels, elevated intracellular Ca^2+^ levels induce CaMKII auto-phosphorylation (Chen et al., 2018). The activation of NMDAR and AMPAR is mediated by recruitment of additional receptors to the postsynaptic membrane and phosphorylation of existing synaptic receptors, in which NR2B and GluR1 have been suggested to play critical roles in regulating nociceptive pathways of the spinal dorsal horn (Luo et al., 2014). During neuropathic pain, following strong synaptic activation, phosphorylation of NR2B at Tyr1472 or Ser1303 increased and induced CaMKIIα auto-phosphorylation at Thr286, then promoted phosphorylation of the GluR1 at Ser831 and enhanced its current (Katano et al., 2011; Zhou et al., 2011). In this study, we found that LPM580098, to some extent, suppressed membrane recruitment of NR2B and GluR1, and inhibited the phosphorylation levels of NR2B subunit (Tyr1472, Ser1303), GluR1 subunit (Ser831), and CaMKIIα (Thr286) after SNL. A plausible interpretation of LPM580098-induced analgesia may be that the increase in the release of presynaptic neurotransmitters (i.e., 5-HT, NE, DA) activates descending inhibitory systems and reduces the release of excitatory neurotransmitters in the spinal dorsal horn, including glutamate (Iacono and Gross, 2008; Tang et al., 2009; Obata, 2017), which then inhibits postsynaptic amplifications of neuropathic pain via NR2B/CaMKIIα/GluR1 signaling pathway.

The functional and structural plasticity of dendritic spines are regarded as visual indicators of synaptic plasticity and memory (Nishiyama and Yasuda, 2015). Neuropathic pain induces dendritic spine remodeling, which occurs 28 days after spinal cord injury in dorsal horn neurons (Kim et al., 2006), leading to alterations in the synaptic structure and an increase in spine density (e.g., total spine density, thin and mushroom-shaped spine density), with underlying input–output neural correlations that are related to nociceptive signaling (Tan et al., 2008, 2011; Tan and Waxman, 2012). As such, we examined the dendritic spine remodeling by using Golgi-stained spinal cord dorsal horn neurons. It was found that the remodeling of dendritic spines, as indicated by an increase in total spine density, thin and mushroom-shaped spine density, occurred after SNL and LPM580098 treatment effectively reduced SNL-induced increase. Our results indicate that LPM580098 inhibits dendritic spine remodeling after SNL in dorsal horn neurons.

Accumulating evidence has established that Rho GTPases, namely, RhoA and Rac1, can modulate actin polymerization and influence the morphology and function of spines (Golden et al., 2013; Wang et al., 2013). Activated Rac1 promotes dendritic spine growth and synaptic strength in dorsal horn neurons, whereas the RhoA-mediated signaling cascade promotes the polymerization of actin filaments, rearranges the cytoskeleton, and allows intracellular trafficking of various nociceptive signaling factors (Ohsawa et al., 2016). Phosphorylated CaMKIIα mediates synaptic transmission, which also regulates the actin cytoskeleton (Okamoto et al., 2009) through activation of Rac1 pathways by phosphorylating the Rac GEFs (Tiam1 and Kalirin-7) and activation of the RhoA/ROCK pathway during spine formation (Tolias et al., 2005; Xie et al., 2007; Penzes et al., 2008; Okamoto et al., 2009). Rac1 inhibitors, which inhibit Rac1 activity after spinal cord injury, reduce the expression of PSD95 (Tan et al., 2008), which is an important scaffold protein in the postsynaptic membrane. The overexpression of PSD95 in the spinal dorsal horn can drive the maturation of excitatory glutamatergic synapses (Sharma et al., 2006). To examine the effect of LPM580098 on dendritic spine structural plasticity, Western blot analysis was performed, which showed that the expression of Rac1/RhoA and PSD95 decreased with LPM580098 treatment after SNL. These results suggest that LPM580098 reduces postsynaptic dendritic spine remodeling through Rac1 and RhoA regulators after SNL.

## Conclusion

In conclusion, our present study provides the first line of evidence that LPM580098, a potential TRI, displays robust analgesic effects in rodent models of persistent pain and neuropathic pain without inducing sedation and somnolence, two sides effects commonly seen in clinical use of first-line analgesics for neuropathic pain such as pregabalin, duloxetine or gabapentin. The potential analgesic mechanism of LPM580098 may be related to its inhibition on presynaptic reuptake of 5-HT, NE, and DA, and suppression of abnormal synaptic plasticity by downregulating the NR2B/CaMKIIα/GluR1 and Rac1/RhoA signaling pathways. Together, our findings strongly suggest that LPM580098 is a promising treatment for neuropathic pain without inducing sedation and somnolence effects and warrants further investigation as a potential analgesic.

## Author Contributions

MY synthesized the compound. CL and JT designed the research work. NL, RH, and YW performed the experiments. NL and CL analyzed the data. NL drafted the manuscript. CL, NL, JT, and HW revised the content of the paper. All authors read and approved the submitted version.

## Conflict of Interest Statement

The authors declare that the research was conducted in the absence of any commercial or financial relationships that could be construed as a potential conflict of interest.

## Abbreviations

5-HT: serotonin
AMPAR: AMPA receptor
CaMKII: Ca^2+^/calmodulin-dependent protein kinase II
CMC-Na: sodium carboxymethylcellulose
CNS: central nervous system
DA: dopamine
DAT: dopamine transporter
GluR: glutamate receptor
NE: noradrenaline
NET: norepinephrine transporter
NMDAR: *N*-methyl-D-aspartate receptor
NMR: nuclear magnetic resonance
PWL: paw withdrawal latency
PWT: paw withdrawal threshold
SERT: serotonin transporter
SNc: substantia nigra pars compacta
SNL: spinal nerve ligation
SNRIs: 5-HT and NE reuptake inhibitors
TRI: triple reuptake inhibitor
VTA: ventral tegmental area
WDR: wide dynamic range.
