# Supplementary figures and images for: LPM580098, a Novel Triple Reuptake Inhibitor of Serotonin, Noradrenaline, and Dopamine, Attenuates Neuropathic Pain
Source: Front Pharmacol. 2019 Feb 14;10:53. doi: 10.3389/fphar.2019.00053 (PMC6382704; doi:10.3389/fphar.2019.00053)

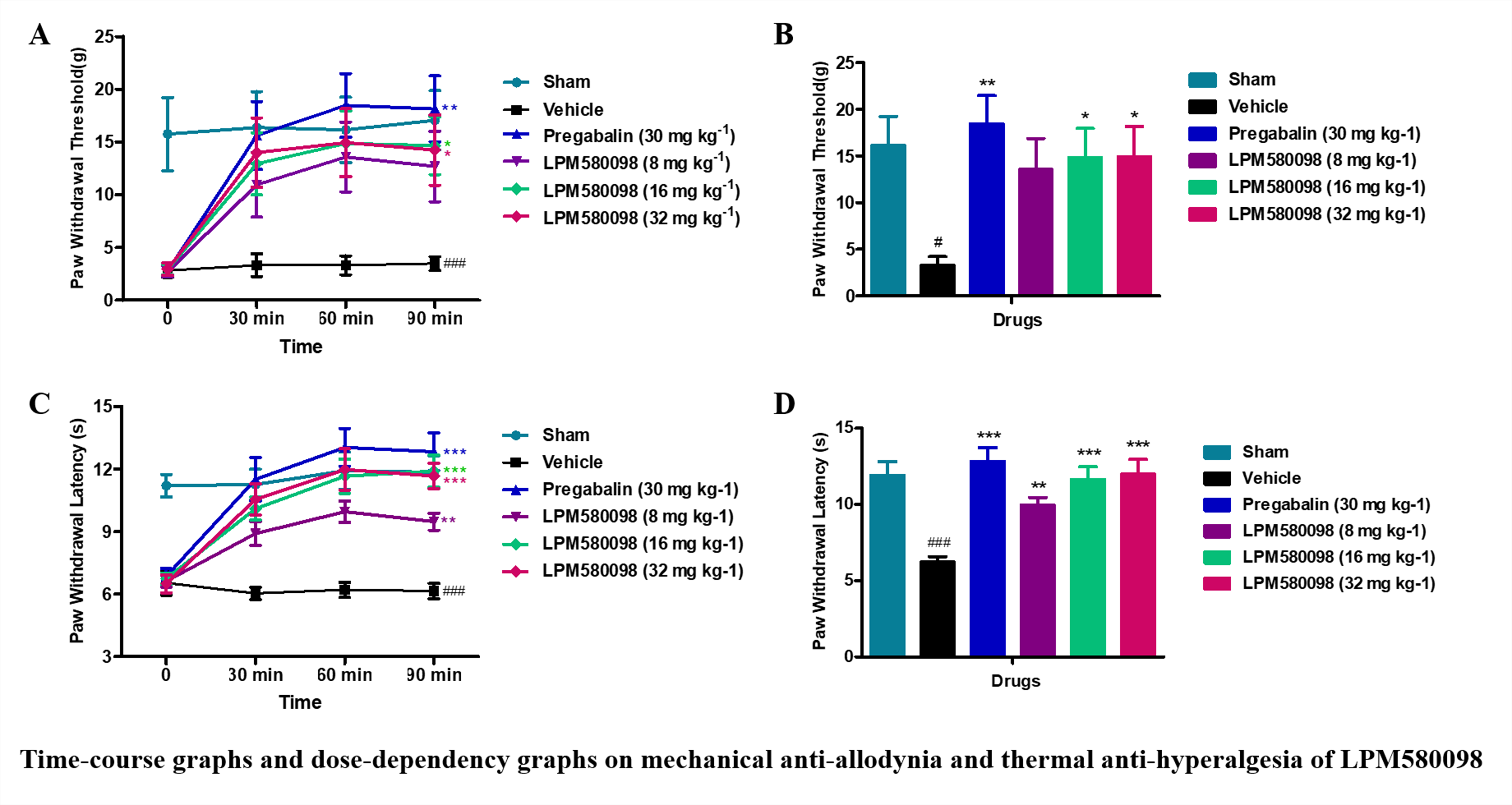

Supplement: Supplementary file 1 [file Image_1.TIF]
