# Supplementary material for: LPM580098, a Novel Triple Reuptake Inhibitor of Serotonin, Noradrenaline, and Dopamine, Attenuates Neuropathic Pain
Source: Front Pharmacol. 2019 Feb 14;10:53. doi: 10.3389/fphar.2019.00053 (PMC6382704; doi:10.3389/fphar.2019.00053)
Supplement: Supplementary file 2 [file Data_Sheet_1.docx]

**Supplementary figure legends and comments**

**Figure. A.** Time course of the analgesic effects of LPM580098 on SNL-induced mechanical allodynia in rats. Von Frey filaments were used to assess mechanical allodynia at 0, 30 min, 60 min and 90 min post administration an oral dose of vehicle (0.5% CMC-Na), pregabalin (30 mg kg^-1^) and LPM580098 (8 mg kg^-1^, 16 mg kg^-1^, 32 mg kg^-1^). Mechanical PWT of the right hind paw significantly decreased in the SNL-vehicle group compared to the sham animals (^###^*p* < 0.001); while LPM580098 (16 mg kg^-1^, 32 mg kg^-1^) and pregabalin (30 mg kg^-1^) treatment significantly increased PWT compared to the SNL-vehicle group (^*^*p* < 0.05, ^*^*p* < 0.05, ^**^*p* < 0.01). Data were expressed as the mean ± SEM, n = 10/group, two-way ANOVA followed by LSD test.

**Figure. B.** The analgesic effects of LPM580098 on SNL-induced mechanical allodynia in rats. Behavioral testing was performed at 60 min after oral administration of vehicle (0.5% CMC-Na), pregabalin (30 mg kg^-1^) and LPM580098 (8 mg kg^-1^, 16 mg kg^-1^, 32 mg kg^-1^). The PWT of SNL-vehicle rats significantly decreased compared to the sham animals (^#^*p* < 0.05); while LPM580098 (16 mg kg^-1^, 32 mg kg^-1^) and pregabalin (30 mg kg^-1^) treatment significantly increased PWT compared to the SNL-vehicle group (^*^*p* < 0.05, ^*^*p* < 0.05, ^**^*p* < 0.01). Data were expressed as the mean ± SEM, n = 10/group, one-way ANOVA followed by Dunnett’s post hoc analysis.

**Figure. C.** Time course of the analgesic effects of LPM580098 on SNL-induced thermal hyperalgesia in rats. Thermal hyperalgesia was assessed at 0, 30 min, 60 min and 90 min post administration an oral dose of vehicle (0.5% CMC-Na), pregabalin (30 mg kg^-1^) and LPM580098 (8 mg kg^-1^, 16 mg kg^-1^, 32 mg kg^-1^). The PWL of the right hind paw significantly decreased in the SNL-vehicle group compared to the sham animals (^###^*p* < 0.001); while LPM580098 (8 mg kg^-1^, 16 mg kg^-1^, 32 mg kg^-1^) and pregabalin (30 mg kg^-1^) treatment significantly increased PWL compared to the SNL-vehicle group (^**^*p* < 0.01, ^***^*p* < 0.001, ^***^*p* < 0.001, ^***^*p* < 0.001). Data were expressed as the mean ± SEM, n = 10/group, two-way ANOVA followed by LSD test.

**Figure. D.** The analgesic effects of LPM580098 on SNL-induced thermal hyperalgesia in rats. Behavioral testing was performed at 60 min after oral administration of vehicle (0.5% CMC-Na), pregabalin (30 mg kg^-1^) and LPM580098 (8 mg kg^-1^, 16 mg kg^-1^, 32 mg kg^-1^). The PWL of SNL-vehicle rats significantly decreased compared to the sham animals (^###^*p* < 0.001). while LPM580098 (8 mg kg^-1^, 16 mg kg^-1^, 32 mg kg^-1^) treatment significantly increased PWL in a dose-dependent manner compared to the SNL-vehicle group (^**^*p* < 0.01, ^***^*p* < 0.001, ^***^*p* < 0.001). Data were expressed as the mean ± SEM, n = 10/group, one-way ANOVA followed by Dunnett’s post hoc analysis.

In conclusion, the results from the preliminary research of LPM580098 suggest that the most appropriate point to detect mechanical allodynia and thermal hyperalgesia in SNL rats is at 60 min after oral administration, and LPM580098 at a dose of 16 mg kg^-1^ was selected for further studies.
